# World Hepatitis Day — July 28, 2013

**Published:** 2013-07-26

Established by the World Health Assembly in 2010, the third annual World Hepatitis Day will be observed July 28, 2013. Viral hepatitis is a leading cause of infectious disease mortality globally, each year causing approximately 1.4 million deaths (1). Most of these deaths occur among the approximately 400 million persons living with chronic hepatitis B virus (HBV) or hepatitis C virus infection who die from cirrhosis or liver cancer years and decades after their infection. In addition to HBV, hepatitis A virus is a leading cause of vaccine-preventable death globally (1). Hepatitis E virus (HEV) also causes significant morbidity and mortality, particularly in Asia and Africa.

HBV and HEV infection are important yet largely neglected causes of maternal and infant morbidity and mortality in resource-constrained settings. This issue of *MMWR* includes a report describing the investigation of a hepatitis E outbreak among refugees in South Sudan, where a significant proportion of affected pregnant women died from HEV infection. A second report from Laos describes missed opportunities for vaccination of newborns to protect them from mother-to-child transmission of HBV.

Prevention of both new infections and mortality from viral hepatitis are the goals of global control efforts. Additional information on viral hepatitis for health professionals and the public is available at http://www.cdc.gov/hepatitis.

## References

[1] Lozano R, Naghavi M, Foreman K (2012). Global and regional mortality from 235 causes of death for 20 age groups in 1990 and 2010: a systematic analysis for the Global Burden of Disease Study 2010. Lancet.

